# USING ANOCRITICISM AS AN ANALYTICAL FRAMEWORK: PERSPECTIVES FROM THE HUMANITIES

**DOI:** 10.1093/geroni/igac059.1487

**Published:** 2022-12-20

**Authors:** Kate de Medeiros, Roberta Maierhofer

## Abstract

To date, Gender Studies and Age Studies (predominantly humanities-based approaches to studying old age) have mostly remained two separate areas of research despite the ontological and epistemological similarities of both fields. As Maierhofer points out, “[a]ge/[a]geing studies would not have been established as a field without the theoretical and methodological approaches established through feminist theory.” Age studies research has largely focused on cultural representations (e.g., novels, films) and social manifestations of either age or gender but not both. Considering this limitation in research, this interdisciplinary panel discusses cultural and social intersections of age and gender from a theoretical as well as an empirical perspective. Taking Maierhofer’s analytical approach of ‘anocriticism’ as a starting point, the contributions of this panel explore intersections of age and gender in society, culture, and cultural representations. The first paper traces the historical development of anocriticism, from its feminist origins to its current application in Age Studies scholarship. The second paper presents research from two studies which used anocriticsm as an analytical framework. The third reports findings from an analysis of two novels. Here, an anocritical lens was used to deconstruct heteronormative assumptions about gender and age. The final paper presents an anocritical, qualitative analysis of narratives from five older men with regarding their performance of masculinities and power. As the papers demonstrated, anocriticism as theoretical framework, allows for a multi-dimensional yet nuanced analysis of cultural and social intersections of age and gender.

